# Fresh and Rheological Performances of Air-Entrained 3D Printable Mortars

**DOI:** 10.3390/ma14092409

**Published:** 2021-05-05

**Authors:** Yeşim Tarhan, Remzi Şahin

**Affiliations:** 1 Department of Civil Engineering, Engineering Faculty, Ardahan University, 75002 Ardahan, Turkey; 2 Department of Civil Engineering, Engineering Faculty, Atatürk University, 25240 Erzurum, Turkey; rsahin@atauni.edu.tr

**Keywords:** 3D printable mortar, air-entraining admixture, fresh properties, rheology

## Abstract

The effect of air-entraining admixture (AEA) on the fresh and rheological behavior of mortars designed to be used in 3D printers was investigated. Blast furnace slag, calcined kaolin clay, polypropylene fiber, and various chemical additives were used in the mortar mixtures produced with Super White Cement (CEM I 52.5 R) and quartz sand. In addition to unit weight, air content, and compressive strength tests, in order to determine the stability of 3D printable mortar elements created by extruding layer by layer without any deformation, extrudability, buildability, and open time tests were applied. Fresh and rheological properties of 3D printable mortars were also determined. It was concluded that the addition of AEA to the mortars decreased the unit weight, viscosity, yield, and compressive strength, but increased the air content, spread diameter, initial setting time, and thixotropy of 3D printable mortar. It is recommended to develop a unique chemical admixture for 3D printable mortars, considering the active ingredients of the chemical additives that affect fresh and rheological performance of mortar such as superplasticizer, viscosity modifying, and cement hydration control.

## 1. Introduction

3D-object production was first developed by Charles Hull in 1984 using numerical information [1]. In this method, a 3D digital model is converted into a stereolithography (STL) format and sent to the 3D printer. Since almost all of the methods are based on building an object layer upon layer, creating a product with a 3D printer was defined in ASTM F2792-12a [2] as Additive Manufacturing (AM). The fourth industrial revolution (Industry 4.0), on the other hand, has been aimed at the digitalization of the most complex industrial products and industrial works. 3D printer technology as an application of digitalization is used in many areas such as industrial manufacturing, medicine and health, aviation and space, architecture and construction, military applications, textiles, food, and education.

Although approaches of construction automation and digitalization are still in the innovation or seed phases, as it has many advantages, AM in the construction industry will be applied to large-scale elements in the near future due to increasing scientific research data and technological developments [3,4]. Some advantages of the method can be exemplified as follows; faster construction, lower building cost, more geometric freedom, shorter supply chain, improved productivity, lower energy consumption, less waste, non-use formwork, safer construction sites, and social benefits such as opportunities for gender equality and construction workers to acquire new skills which include the use of robotic systems [5,6,7,8,9,10,11,12,13,14,15,16,17,18,19]. Besides all these benefits, sizes of 3D printers, directional dependency, cybersecurity, interoperability, and lack of standards and regulations are among the disadvantages of 3D printing in the construction sector [9,11,15,18,20,21,22,23,24,25,26,27].

Mortar/concrete that can be used in the 3D printer must be extruded to an acceptable degree so that it can be removed from the nozzle of the printer, must have sufficient buildability properties, must be rigid to support other layers without collapsing, and finally, must have sufficient time (open time) for maintaining workability [10]. The effort to meet all the mentioned properties at the same time makes the 3D printable mortar or concrete mix design very complicated.

It is well known from the literature (e.g., [28,29]), on the other hand, that air-entraining additives (AEAs) improve concrete resistance against frost attack. AEAs consisting of surface-active agents or surfactants reduce surface tension at the water/air interface and decrease the damaging effect of the hydraulic pressure resulting from freezing–thawing cycles of concrete due to the intentional creation of tiny air bubbles caused by soluble salts, wood resins, stearic acid, and lignosulfonate acid [28]. As stated in [30], AEA can be used to produce massive micro-pores (50–1.250 μm) with a uniform pore shape and reduce the liquid–air interfacial tension with improved hydration shell thickness. For a material with high yield stress such as fresh concrete, the extra air-entraining agent can be added to decrease the rheological parameters for better pumpability [31]. In addition, adding air-entraining admixture is one of the effective methods to reduce the density of fresh concrete, and by reducing the concrete density, the layers below will be able to easily carry the layers added one on top of the other [9,32]. Lu et al. [33] designed spray-based 3D printable cementitious materials with fly ash cenosphere (FAC) and air-entraining agents and used these materials for density reduction of concrete. Assaad et al. [34] compared the efficiency of AEA and styrene-butadiene rubber (SBR) latexes to protect 3D printable mortars against deterioration due to frost attack and determined that the incorporation of SBR was more efficient than AEA to reduce the drop in bond strength due to freeze–thaw cycles. They also indicated that air entrainment would only protect the layer itself against frost, while the interface between successive layers remains vulnerable to frost attack and risks of delamination. Das et al. [35] studied the effect of different processing (pumping, acceleration/mixing, and extrusion) encountered in 3D printing of mortar and emphasized that the effect of the processing conditions on the stable air-void system resulting from the AEA should be taken into account.

Considering the current state of 3D printable materials, it can be seen that there is still not enough focus on material properties. De Schutter et al. [36] reported that currently available high-performance cement-based materials cannot be directly 3D printed due to their inadequate rheological and stiffening properties. Rahul et al. [37] stated that despite its rapid growth, there is only a limited understanding of the material requirements for 3D printability. According to Buswell et al. [38], 3D concrete printing (nowadays often referred to as 3DCP) manufacturing processes, which require expert machine operators and extraordinary care in the preparation and formulation of materials, are currently inconsistent and unreliable. Wangler et al. [39], on the other hand, stated that material challenges are significant to control early age hydration, rheology, and structural and durability performance, and concluded that material selection is the main issue for mechanical design of 3D printable concrete. Marchon et al. [40] also reported that one of the main issues for hydration and rheology control of concrete for digital fabrication is the material properties. For extrusion-based large-scale digital construction, cement-based materials need to exhibit optimum rheological and mechanical properties to comply with often conflicting requirements such as pumpability, extrudability, and buildability [41]. Bos et al. [42] reported that the material characteristics are an important (although not sole) parameter to determine the buildability of 3D printed concrete; however, they stated that it is not yet clear which material properties are the most suitable for 3D printable cementitious mortars, despite some suggestions that have appeared in earlier works. These results expressed by the researchers also reveal the importance of studies on the determination of material properties for three-dimensional-printed mortars or concretes.

Most of the researchers studying the topic provided valuable suggestions and conclusions about the constituents that make up the binding phase (matrix) of 3D printable mixes. For example, Le et al. [43], Jeon et al. [44], Hambach et al. [45], Rahul et al. [37], Kazemian et al. [46], Panda and Tan [47], and Panda et al. [48] determined that traditional mineral admixtures such as fly ash, silica fume, and ground granulated blast furnace slag increase the performance of 3D printable concretes. Marchon et al. [40] stated that it is essential to take into account inorganic additives such as fly ash, slag, and silica fume or calcined clay for 3D printed concrete mix design to obtain easily extrudable mixtures. Srinivasan et al. [49] and Kuder and Shah [50], on the other hand, indicated that rheological modifiers like calcined clay were found necessary for a successful extrusion. Tregger et al. [51] studied the effect of calcined clay, fly ash, and high-range water-reducing admixture on the green strength of cement paste. Voigt et al. [52] searched the effect of fly ash and calcined clay on flowability and shape stability of 3D printed concrete. Panda et al. [53] used high volume fly ash mixtures with the nano-attapulgite clay to improve the printability of 3D concrete. Kondepudi and Subramaniam [54] studied a baseline mixture in which alkali-activated fly ash and slag were modified using dry components such as micro-silica and clay. Other researchers (e.g., [55,56,57]) have also noted that clays can be used as rheological modifiers for cement-based materials, as well-chosen additives such as clay powders and chemical admixtures help to achieve the desired level of thixotropic behavior in 3D concrete while on the move. On the other hand, the number of studies in the literature investigating the effect of air-entraining admixture on the fresh properties of 3D printable mortar is quite limited, and some of the findings in the studies contradict each other [33].

The first part of a comprehensive scientific research [58] whose main purpose was to investigate the effect of AEA on the behavior of 3D printable mortars in fresh and hardened state is presented in this article. Another aim of this study was to contribute to the content of special chemical additives to be produced for 3D printable mortar. For this purpose, mortars were produced using all chemical additives proved by preliminary experiments that they can contribute positively to the improvement of the basic characteristics of 3D printable cement-based materials. It is expected that the results of the study will also contribute to the clarification of the contradicting findings in the literature regarding the addition of AEA to 3D printed concrete.

## 2. Experimental Methodology

### 2.1. Materials

Rapid hardening Super White Cement (CEM I 52.5 R in accordance with EN 197-1:2011) and ground granulated blast furnace slag (GGBFS) formed the binder components. Super White Cement was chosen for its superior adhesion strength and high strength [43,59], while GGBFS was determined as a mineral additive to both increase the performance of 3D concrete and to be compatible with white cement [43,60,61]. Due to its high calcium content, the fast-setting time of GGBS at room temperature was also considered an advantage. Additionally, high purity calcined kaolin clay was preferred as a rheology regulator to improve shape stability and printing quality due to its water-retaining property [62,63,64]. Chemical compositions and physical properties of the three components are shown in Table 1.

Two different fine aggregate classes of silica sands with sizes of 0–0.5 mm and 0–1 mm and particle densities of 2.44 and 2.49 respectively were used in the mixtures. Monofilament synthetic (polypropylene: pp) microfibers (commercially available as MasterFiber M 100) which are an ultra-thin polypropylene fiber with high tensile strength, high elasticity modulus, designed to disperse quickly and homogeneously throughout the mortar matrix were added to mixtures to reduce shrinkage and crack formation. The fibers were 13~19 mm in size and had specific gravity of 0.91, tensile strength of 480 MPa, and modulus of elasticity of 8.48 GPa.

Based on the evaluations in terms of criteria such as consistency, setting time, 3D concrete characteristics of the mixtures, and flowability from the nozzle of a 3D printer, many preliminary experiments were carried out within the scope of the study. According to the findings obtained from the preliminary tests given in [58], we decided to add more than one chemical additive to the mixtures. High-performance viscosity modifying agent (VMA1, commercially available as MasterMatrix^®^ SDC 100) and superplasticizer (MasterGlenium^®^ T 803) were used in order to provide the extrudability and buildability properties of 3D printed mortar and to regulate the workability. Due to the early setting property of CEM I 52.5 R-type cement and the effect of VMA1, 3D printable mortars began to harden within minutes while they were in fluid consistency when they were first poured. Therefore, a non-chloride chemical admixture (MasterRoc^®^ HCA 20) was used to control the dynamics of cement hydration and the workability time of the mortar. Another viscosity modifying agent and strength enhancer (VMA2) (MasterRoc^®^ MS 685) was needed to provide improved cohesion, reduce porosity, and increase the compactness of mixtures. In order to ensure the buildability of 3D printable mortars, the layers added on top of each other must start to set after “a certain period of time” in order to carry each other. A setting accelerator additive (MasterRoc^®^ SA 194) was added to the mixtures as the cement hydration control as admixture shortened the period too much. Since the amount of entrained air was chosen as a parameter in the experimental study, air-entraining admixture (MasterAir MA 1) was also added to the mixtures. Finally, when used with the air-entraining admixture, a high-performance plasticizer/set retarding additive (MasterSet R 2) was also used in accordance with the manufacturer’s recommendation as it improves flowability and workability of the mixture. Table 2 shows the specifications provided by the manufacturer (Master Builders Solutions Yapı Kimyasalları, İstanbul, Turkey) of all chemical additives used in the mixtures.

### 2.2. Proportions of Mortar Components, Mix Design, and Coding

The rates of air-entraining admixture were selected at 4 different levels as 0, 0.1, 0.15, and 0.2% of the binder amount. The water/binder ratio in this study was kept at 0.35 for all the mixes. Cement dosage was also kept high (680 kg/m^3^) in order to improve the workability properties and to increase the fluidity from the nozzle of a 3D printer. The percentage of blast furnace slag was determined as 20% of the cement weight and was used by adding to the cement dosage (i.e., the total binder amount was 828 kg/m^3^). Based on preliminary studies, the microfiber rate was decided as 0.2% of the whole mixture volume. In the mixtures, the aggregates with maximum aggregate size (D_max_) of 1 mm were 2 times the aggregates with D_max_ of 0.5 mm by volume and the total aggregate amount was 1.24 times the amount of binder.

Mix design for the four groups produced within the scope of the study is given in Table 3. In the study, the groups containing AEA at the rates of 0, 0.1, 0.15, and 0.20% were coded as, respectively, A0, A1, A1.5, and A2.

### 2.3. Test Procedures

Since there are no standardized methods for determining the technical specifications of mortar or concrete mixes for 3D printing, many researchers have proposed their own methods for laboratory testing of printable cementitious materials [65]. The fresh state properties of all mixes were examined both by the conventional fresh concrete tests (consistency, unit weight, and air content) and by the interrelated characteristics (extrudability, buildability, and open time) which were necessary for proper extrusion and forming of 3D printable mortar in this study. Tests for determining the rheological behavior of all mixtures and compressive strength after curing were also made during the experiments. In the study, as with all types of concrete, the compressive strength tests of 3D printed mortars were also carried out, as they provided useful information about both durability and mechanical properties of the mortars.

#### 2.3.1. Experimental Procedure of Preparation of the Samples

The detailed procedure of preparation of the 3D printed mortars was as follows: firstly, all dry and powder materials (cement, GGBFS, aggregates, clay, and microfiber) were added to the mixture and mixed in a mortar mixer at low speed (62 rpm) for one minute, then half of the water was added to the mixture and also mixed at a low speed for one minute. After these procedures, superplasticizer, cement hydration control, viscosity modifyer, setting accelerator, viscosity modifying and strength enhancer, and plasticizer/set retarding admixtures were added respectively and separately to the mixture, together with the remaining water and mixed at moderate speed (140 rpm) each one for one minute. The mixture was stirred for one more minute at high speed (285 rpm) and rested for one minute. Finally, AEA was added to the mixture and mixed at high speed for only one minute and pouring process of the mortar was started. In total, the mixing time was approximately 11 min.

#### 2.3.2. Consistency, Unit Weight, and Air Content of Fresh Mortar

To determine the fresh properties of the 3D printable mortar, the following tests on the mixtures were performed:

- Determination of consistence of fresh mortars (by flow table) in accordance with TS EN 12350-5 [66], ASTM C 230 [67], and ASTM C 1437 [68];
- Unit weight of fresh mortar in accordance with TS EN 12350-6 [69];
- Determination of air content of fresh mortar in accordance with TS EN 12350-7 [70].

Based on the research results by Lachemi et al. [71] and Ma et al. [72], the flow table test was used to evaluate the viscosity of fresh mortar and the deformation through restricted areas. Since it was determined in previous studies (e.g., [73]) that the air-entraining additives reduced the unit weight of the concrete, it was necessary to control the unit weights of the mixtures. On the other hand, since the main parameter of this study was the air-entraining admixture, an air content test was required to determine the amount of air obtained by entraining air into the fresh mortar produced in the experiments.

#### 2.3.3. Extrudability

**Figure 1:**
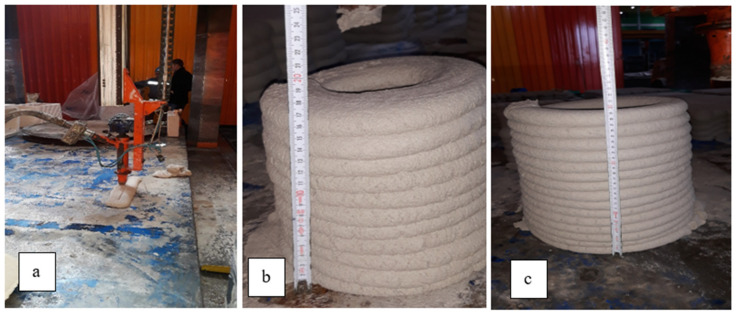
Determination of the extrudability and buildability properties of fresh mortars before production of full-size samples (mono-(**a**) and multi- (**b**,**c**) layer casting).

3D printable cement-based materials must be extrudable in structural integrity without discontinuity and segregation, maintaining consistency throughout the entire casting process [65]. As stated in the studies by Zhang et al. [18], Rahul et al. [37], and Kazemian et al. [46], the fact that the overlapping mortar layers, which can be easily poured from the pump end, have the same thickness and height everywhere during the pouring shows that mortar has inline quantification of extrudability. During the experiments carried out in this study, it was observed that the thicknesses of the layers were the same everywhere in the measurement made every 10 cm along the 30 cm line (see Figure 1a).

#### 2.3.4. Buildability

Buildability is an indicator of the feasibility of fresh mix for additive printing and the resistance to deformation under the pressure of subsequent layers. This characteristic can be determined by measuring the maximum level at which poured mortar can be climbed without crushing and collapsing [74]. In this study, it was determined that the crushing started on the lowest layer after reaching approximately 10 layers with pouring a circle of mortar (see Figure 1c) and thickness of each layer was approx. 2.5 cm (see Figure 1b).

#### 2.3.5. Open Time

Open time, also known as printability, is a period in which the mix maintains proper pumpability and in this period the mix must maintain desired quality and adhesion in a layer-by-layer structural build-up [65]. Open time of the 3D printable cementitious materials can be determined based on the shear stress test, jump table, Vicat apparatus, V-Funnel method, penetration tests, and mini cone. In this study, the open times of the mixtures were determined with the Vicat apparatus, considering the suggestions made by the authors of [43,72,75]. In the experiments, 3D printable mortars were filled in the Vicat mould specified in TS EN 196-3 [76] without rodding then the penetration depths of the needle were measured at certain time intervals and the open times of the mortars were interpreted by using these measurements.

#### 2.3.6. Rheological Properties

Rheology is a discipline that studies the deformation and flow properties of a material under stress and provides a better understanding of the properties of fresh cement-based materials. The most suitable model that represents rheological behavior of fresh mortar and concrete is the Bingham model [77] represented by the following equation:

τ = τ_0_ + μγ (1)

Here τ (Pa) defines the shear stress at γ (1/s) shear rate, and τ_0_ (Pa) and μ (Pa.s) define, respectively, the shear threshold and plastic viscosity.

Based on the model, it can be said that each fresh mortar mix has a threshold shear value and plastic viscosity. The shear threshold (τ_0_) is the shear stress required to initiate flow applied to a material. When the shear stress exceeds the shear threshold, the material starts to flow and the resistance to flow depends on the plastic viscosity. Plastic viscosity, on the other hand, refers to the resistance of the material against flowing after it exceeds the slip threshold. Rheological properties such as viscosity and shear threshold can be measured in cement paste, mortar, and concrete with a rheometer using the Bingham model [78,79,80]. In this study, a rotational rheometer (trade name: RheolabQC, a product of Anton Paar GmbH, Graz, Austria) was used to determine the viscosity properties of 3D printable mortars.

## 3. Results and Discussion

**Figure 2:**
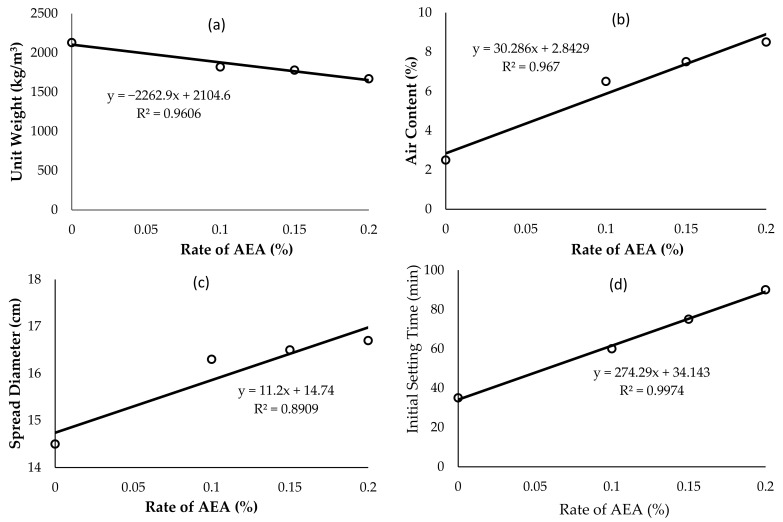
The effect of rates of AEA on the unit weight (**a**), air content (**b**), spread diameter (**c**), and initial setting time (**d**) of 3D printable mortar.

The results of all the fresh and hardened 3D printable mortar tests are given in Table 4. Based on the data given in Table 4, the graphs showing the behavior of fresh mortar are given in Figure 2 and the experimental findings given in the table are evaluated below.

### 3.1. Evaluation of Unit Weight Tests Results

The results of the unit weight test of 3D printable mortar mixtures are plotted in Figure 2a. As can be seen from the figure, the unit weights of the 3D printable mortars linearly (R^2^ = 0.9606) decreased with increasing the dosage of the air-entraining admixture in the mixtures. These decreases brought the unit weight of 3D printable mortars lower than those of normal weight concrete, and even to the upper limits determined in TS EN 206 [81] for lightweight concretes. While the unit weight of A0 group without AEA was found to be 2130 kg/m^3^, the unit weight of A2, which was the group with the highest rate of AEA, was found to be 1670 kg/m^3^. Lu et al. [33] also reported that the unit weights of spray-based 3D printable cementitious materials decreased below 2000 kg/m^3^ with the addition of air-entraining additives. However, the layers in all mortars, including A2 which was the lightest and has the lowest compressive strength, easily carried both their own loads and the loads of the upper layers during production of full-size samples. Therefore, it should be emphasized that all groups have the buildability properties of 3D printable mortar.

As can be seen from the mix design given in Table 3, the cement dosages of the mixes were very high (680 kg/m^3^) compared to conventional mortars/concretes. It was thought that the unit weight loss caused by air entrainment to the mortars produced in the study was caused by the high volume of air created by the air-entraining additive in the mixtures with very high cement dosages.

### 3.2. Evaluation of Air Content Test Results

The findings obtained from the air content tests of 3D printable mortar are graphed in Figure 2b. In this study, it was determined that mortars without air-entraining additives also have an air content of 2.5% (see Table 4), due to the combined effect of fibers and other chemical additives. As can be seen from Figure 2b, on the other hand, increasing AEA dosage caused significant increases in the air contents of mortars. In fact, although the air content of the group without AEA was 2.5%, this ratio reached 6.5% even with the addition of AEA at the minimum dosage (0.1%). However, the increase in the air content of mixtures containing 0.15 and 0.2% AEA was less than that of 0.1% AEA. As a matter of fact, the air content of the mortar group containing 0.1% AEA was 160% higher than the group without AEA, while the air contents of the groups containing 0.15 and 0.2% AEA were 15 and 31% higher, respectively, than the group with 0.1% AEA.

It has been determined in many previous studies that the air-entraining admixture increases the air content of the mortar or concrete. For example, Şahin et al. [82] used AEA at the rates of 0, 0.05, and 0.1% and obtained air quantities between 0–6% in fresh concrete. In a study conducted by Şahin [83], AEAs with different chemical compositions were added to concrete in different proportions (0, 0.00625, 0.0125, 0.025, 0.05, 0.1, and 0.125%) and air contents varying between 1 and 7.5% were obtained. Similar results were reported by Zhang and Ansari [84]. According to TS EN 206 [81], on the other hand, the recommended air content amount to produce concrete resistant to freeze–thaw attack (environment effect degree = XF) is at least 4%. From the values given in Figure 2b, it was concluded that the amount of entrained air produced within the scope of this study was at a sufficient level to produce mortar resistant to the freeze–thaw effect.

Hydrophilic ends of air-entraining agents, which are generally composed of hydrophilic ends attached to a hydrophobic chain, create airspaces by attaching to either cement–water or air–water interfaces [85]. This adsorption greatly reduces the air-water surface tension so that the air-entraining additives achieve the formation and stabilization of small bubbles [86]. From a rheology perspective, entrained bubbles may play a role in the lubrication of the cement paste and increase its volume depending on the specification of the AEA, as a result of which the workability of cement-based materials may increase [87].

In the literature, there are studies with different results regarding the relationship between air content of mortar/concrete and its rheological behavior. For example, Szwabowski et al. [88] determined that while the yield stress and plastic viscosity continued to decrease until the air content of the self-compacting concrete reached 5%, the spreading increased and then remained stable. However, Banfill [78] found that the increase in air content of concrete strongly reduced the plastic viscosity of concrete, but its effects on yield stress were not significant. Barfield and Ghafoori [89] analyzed the performance of concretes made with different AEA types and indicated that fresh concretes with similar air content may show a big difference in slump. Based on these findings, it can be said that there is not always a definite relationship between the air content and rheological properties of fresh mortar and fresh mortar may exhibit different rheological behaviors. As a matter of fact, as will be detailed in the following section, although the air amount of the mortars produced within the scope of this study increased significantly, the spread diameters of the mortars did not change much.

### 3.3. Evaluation of Flow Table Test Results

The flow table test results obtained from the experiments performed on fresh mortars are given in Figure 2c. As can be seen from the figure, the groups with AEA had very close spread diameters (approx. 16 cm), while the group without AEA flowed less (14 cm) than the others. The data generated in this test indicated that adding AEA to mortars without AEA will increase their fluidity but increasing the AEA dosage in mortars with AEA does not have much effect on the increase of the flowability of the mortar. This result was similar to the change in air content caused by the addition of AEA to mortars.

Rahul and Sanatham [90] found the spread diameters of 4 groups of 3D printed mortar to be 18.3–18.7 cm. Lu et al. [33] obtained spread diameters of 3D printable cementitious materials ranging from 15 to 25 cm depending on the ratio of air-entraining admixture. Tay et al. [91] tried to determine the printability zone for 3D printable concrete using the flow table test and found that the spread diameters of 16 groups of the concretes ranged from 11 to 21 cm. Rubio et al. [92], on the other hand, produced concretes with spread diameters of 3D printed concrete ranging from 22 to 28 cm, depending on the ratio of silica fume, fly ash, and polypropylene fiber in the mixtures.

### 3.4. Evaluation of Initial Setting Time Results

As can be seen from Figure 2d, AEA has been very effective in extending the initial setting time of 3D printable mortars. The group without AEA started setting as early as 35 min. On the other hand, by adding AEA to the mixes, the initial setting time of the mixtures was linearly (R^2^ = 0.9974) extended depending on the dosage of the AEA. However, although the initial setting times of the Super White Cement (CEM I 52.5 R) and GGBFS selected for the experiments were 110 and 170 min, respectively (see Table 1), even the initial setting time of the group with the highest AEA ratio (0.2%) did not exceed 90 min. The initial setting time of mortars with and without AEA was shortened by the addition of chemical additives, especially the set accelerating, to the mixtures in order to give 3D mortar characteristics.

Le et al. [43] measured the setting time of high-performance 3D printable concretes using a Vicat apparatus to determine the open time and stated that determining the initial and final time of the setting was not sufficient alone to find the printability time of 3D printable mortars. The observations made in this experimental study also confirmed the results stated in [43], Because, when they are immobile (i.e., if mixing is not continued), 3D printable mortars may begin to lose their workability and solidify even though they have not yet started to set. In other words, if the mortars are not mixed, they may lose their extrusion capability from the nozzle even though they have not hardened yet. This situation leads to questioning the accuracy of the measurements based only on the penetration depth of the Vicat needle.

### 3.5. Evaluation of Compression Strength Test Results

**Figure 3:**
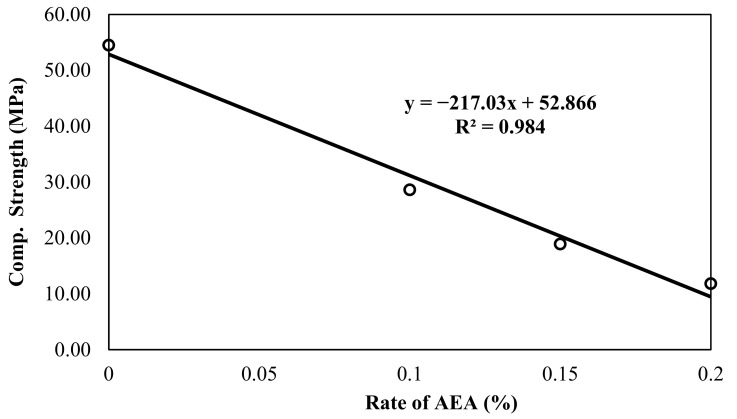
Change of compressive strength of 3D printable mortar with the amount of AEA.

The 28-day compressive strengths determined under uniaxial compression in the 5 × 5 × 5 cm^3^ samples of the 3D printable mortars produced on the basis of the mix design are shown in Figure 3.

In the group without AEA, a compressive strength value (~55 MPa) corresponding to the lower limit of high-strength concrete determined by ACI PRC-363-10 [93] was obtained. In the study conducted by Özalp et al. [75], White Portland 52.5 R-type cement was used and the compressive strength of the 3D printable concrete produced was also close to the strength value found in this study (approx. 60 MPa). However, the compressive strength of the 3D printable mortars decreased dramatically and linearly with the addition of AEA to the mixtures. The amount of reduction compared to the A0 group was 47, 65, and 78% for the A1, A1.5, and A2 groups, respectively.

The reduction in the compressive strength of mortar/concrete with the addition of AEA is an expected result in concrete technology, because, as stated in Şahin et al. [82], billions of small, closed, and independent air voids entrained into concrete by means of AEA cause a decrease in the compressive strength of concrete.

It was observed during the compressive strength tests that plastic deformation on all 3D printable mortars seemed to indicate ductile fractures due to the polypropylene fibers added to prevent shrinkage of the mortars.

**Figure 4:**
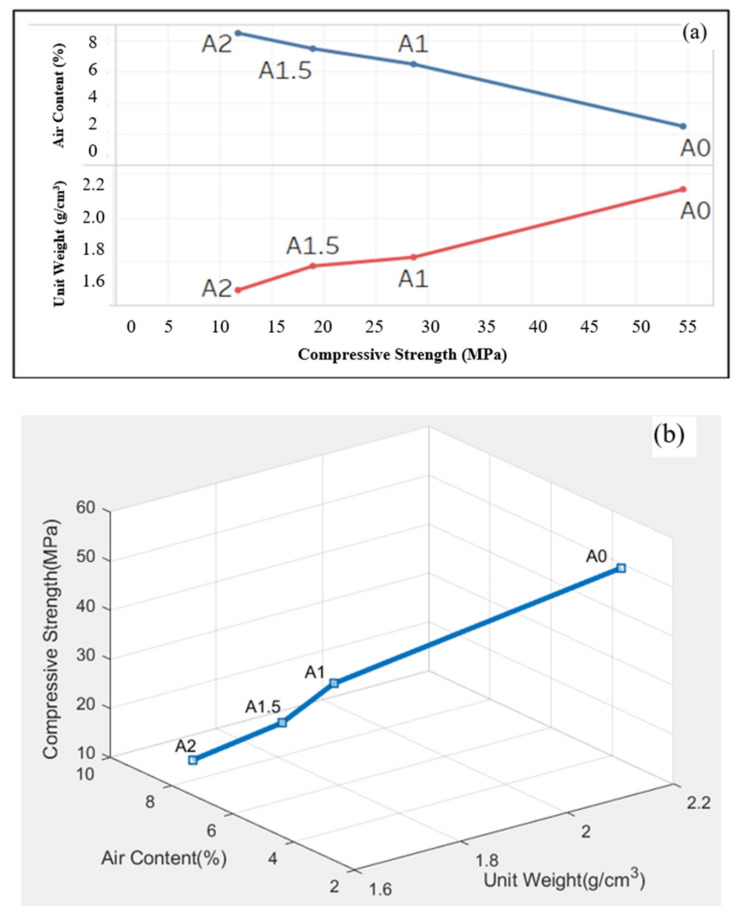
The triple relationship between compressive strength, unit weight, and air content of the mortars on two- (**a**) and three- (**b**) dimensional graphs.

On the other hand, in order to evaluate the relationship between fresh and hardened mortar properties together, the interrelation of the unit weight, air content, and compressive strength of 3D printed mortars is shown in Figure 4.

From Figure 4a, it can be seen that the change of the unit weights and the air contents of the mortars with the compressive strength were roughly mirrored images of each other, with only subtle differences. On the other hand, as can be seen from Figure 4b, it can be observed that there was a relationship between the fresh and hardened properties of the mortars, and the compressive strength of the mortars decreased with the increase of air content and decrease in the unit weight. In other words, in parallel with the literature, the compressive strength of the mixes increased as the unit weights of mortar increased, whereas the compressive strength decreased as the air content of the mortar increased. As a matter of fact, in this study, it was determined that the control group (A0) with the highest unit weight (2130 kg/m^3^), but the lowest air content (2.5%), had the highest compressive strength.

### 3.6. Evaluation of Rheological Properties of 3D Printable Mortar Mixtures

**Figure 5:**
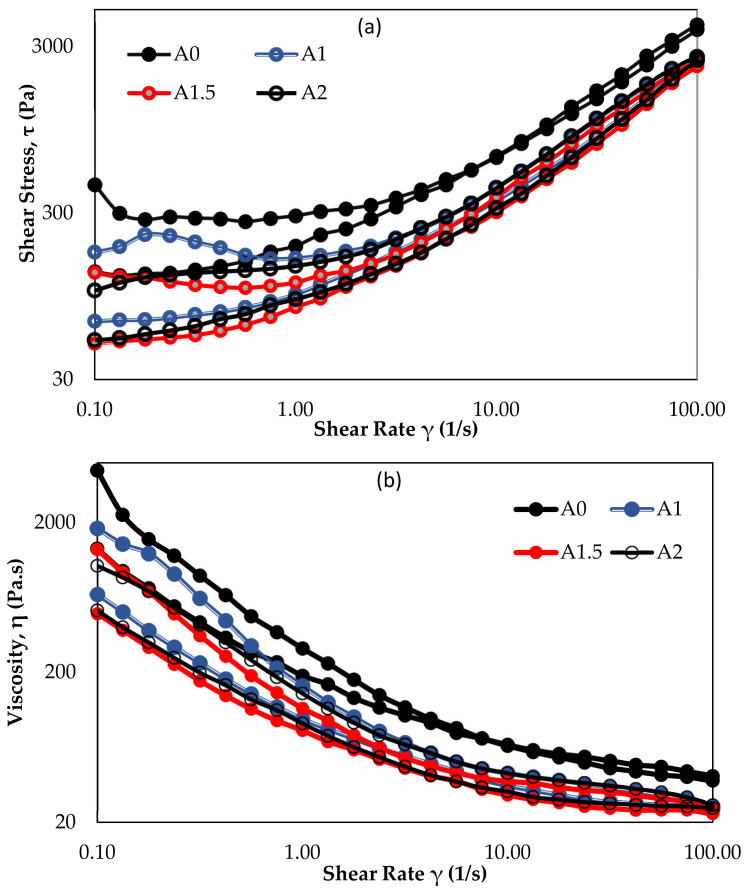
The change of hysteresis loop (**a**) and viscosity (**b**) with the rates of AEA.

During the experimental studies, the shear stress and viscosity of all mixtures were also measured in order to be used in the evaluation of the rheological behavior of 3D printable mortars. The graphs showing the change of shear stress and viscosity of all groups with shear rates are given in Figure 5.

As seen in Figure 5, the shear stress and viscosity of the A0 groups were higher than those of the other groups. Although the air contents of the other three groups (A1, A1.5, and A2) were different (Table 4), their shear stress and viscosity of the groups were very close to each other. However, while the A2 group with the highest AEA was expected to have the lowest viscosity and shear stress, the A1.5 group was the group with the lowest rheological properties.

Similar to the graph given in Figure 2a, the viscosity of 3D printable mortars decreased with the addition of AEA to mixtures compared to those without air-entraining admixtures and the decrease in viscosity of the mixtures continued in parallel with the increase in the AEA amount (see Figure 5b).

Since both the opening time and the extrudability of 3D printable mortars were directly related to the rheological behaviors, yield stress and viscosity of the mixtures were determined and given in Table 5. In the table, the parameters determined during the transition of the device from low shearing speeds to high-shearing speeds are shown as “up (or acceleration ramp)”, and the parameters determined during the transition from high-shearing speeds to low shearing speeds are shown as “down (or deceleration ramp)”. The hysteresis loop method, on the other hand, consists of determining the combination of the up and down flow curves [94], and the area between the rising and falling curves is used as the thixotropy index. Repeating this test at various time intervals can be used as an indicator of structure kinetics [95]. Thixotropy values obtained from the area between hysteresis loops within the scope of this study are also given in Table 5.

**Figure 6:**
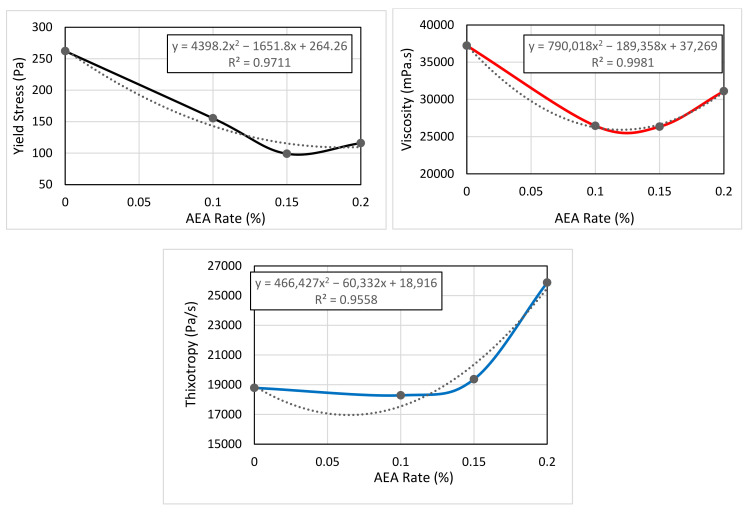
The effect of rates of AEA on the yield stress (**a**), viscosity (**b**), and thixotropy (**c**).

Based on the data in Table 5, the variation of the viscosity, yield stress, and thixotropy of mortars with AEA is given in Figure 6, as they were considered as critical fresh concrete properties to control the printability—as a combination of pumpability, extrudability, and constructability—of 3D printable cementitious materials [95].

The yield stress and viscosity values measured within the scope of this study decreased with increasing the amount of AEA, as can be seen in Table 5 and Figure 6a,b. In other words, as the amount of AEA increased, the concrete became more fluid.

Thixotropic behavior means that the shear resistance of fresh concrete or mortar decreases overtime at a constant deformation rate, and it is the situation that the concrete maintains its fluidity without hardening as long as the mixing process continues. Thixotropy values can also be used for describing the shape stability of the fresh mixture. As can be seen from the results given in Table 5, the thixotropy values of the samples coded as A0, A1, and A1.5 were very close to each other, but the mixture coded as A2 had a higher thixotropy value than those of other groups.

On the other hand, it can be seen from the line of best fits given in Figure 6 that the yield stress and viscosity of the mortars decreased while the thixotropy of the mixes increased with the increase of the AEA ratio. The viscosity, yield stress, and thixotropy values obtained from rheological measurements in this study were compatible with the rheology values of high thixotropic concretes in the literature [43,95,96,97,98,99].

## 4. Conclusions

The main conclusions drawn from the evaluation of the findings obtained from this experimental study are summarized below:

- It is recommended to develop a unique chemical admixture for 3D printable mortars, considering the active ingredients of the chemical additives that affect the behavior of fresh mortar such as superplasticizer, viscosity modifying and cement hydration control.
- Increasing the amount of entrained air in fresh 3D printable mortar mixture by adding AEA increased the air content, but decreased the unit weights of the mixtures, as in conventional mortar or concrete. However, although the amount of air content of the mixes increased, the spread diameters of the mortars did not change significantly.
- Although the initial setting time of the group without AEA (A0) was very short (35 min), the initial setting times of 3D printable mortars increased with the addition of AEA and the group with the highest dosage of AEA (A2) started to set after 90 min. However, even 90 min is lower than the initial setting times of Super White Cement (CEM I 52.5R) and GGBFS selected for the mixtures. As a result of the combined effect of many chemical additives that were chosen consciously in order to acquire the most appropriate mix design for the interrelated characteristics (extrudability, constructability, and open time) determined for 3D printable mortar in the literature, the initial setting times of the mixtures decreased.
- Increasing the dosage of AEA dramatically reduced the 28-day compressive strength of 3D printable mortars. The reductions in compressive strength were, respectively, 47.5, 65, and 78% for the A1, A1.5, and A2 groups compared to that of A0 group. Therefore, it is recommended to pay attention to the use of air-entraining additives in 3D mortar or concrete applications where compressive strength is an important priority.
- The addition of AEA to 3D printable mortars reduced the viscosity and shear stress of the mixtures, and the A1.5 group had the lowest values. Yield stress varying between 50 and 262 Pa was obtained in the study and these values were found to be sufficient for the printability of 3D printable mortar mixes. The thixotropy values of the samples without AEA (A0) and containing AEA at low dosage (A1 and A1.5) were very close to each other, but mixtures containing the highest dosage of AEA (A2) had higher thixotropy values than the other groups.

## Figures and Tables

**Table 1:** Chemical compositions and physical characteristics of the cement, GGBFS, and kaolin clay.

	CEM I 52.5 R	GGBFS	Kaolin Clay
**Chemical Compositions** (**%**)			
SiO_2_	21.21	37.40	49–55
Al_2_O_3_	3.86	10.38	42–46
Fe_2_O_3_	0.32	1.30	0.7 max
CaO	65.85	30.93	0.5 max
MgO	1.05	7.21	0.3 max
SO_3_	3.51	0.77	-
Na_2_O	0.20	0.39	0.2 max
K_2_O	0.49	0.67	0.1 max
TiO_2_	-	0.85	0.8 max
Loss of ignition	3.30	-	0.5 max
Klor (Cl^−^)	0.01	0.0160	-
**Physical Characteristics**			
Initial setting time (min)	110	170	-
Final setting time (min)	130	230	-
Volume expansion (mm)	1	0	-
Specific Gravity (g/cm^3^)	3.04	2.88	2.65–2.75
Bulk density (kg/L)	-	-	0.3–0.5
Spec. Surf. Area (cm^2^/g)	4650	4183	-
Fineness 45 µm (%)	1	1.3	-
Particle size (<2 µm, %)	-	-	83–86
Screen res. (325 mesh, %)	-	-	0.005 max
Whiteness	85.5	-	93.5–94
Comp. Str., 2 days (MPa)	37	-	-
Comp. Str., 3 days (MPa)	43	-	-
Comp. Str., 7 days (MPa)	50	55.3	-
Comp. Str., 28 days (MPa)	60	74.2	-
Oil Absorption (g/100 g)	-	-	50–60
Moisture (105 °C, %)	-	-	0.5 max

**Table 2:** Technical properties of the chemical additives.

NO	Effect	Spec. W. (kg/L)	pH	Color	Cl Ion. (W., %)	Service Tem (°C)	Dosage (%)
I	Viscosity modifying (VMA 1)	1.01 ± 0.01	7.5 ± 1.5	Brown liquid	<0.10	-	%0.1–1
II	Superplasticizer	1.084 ± 0.02	4–5	Dark brown	<0.10	-	0.8–1.5
III	Cement hydration control	1.10 ± 0.02	<2	Red	<0.10	-	0.2–1
IV	Viscosity modifying and strength enhancer (VMA 2)	1.13	9.4	Changeable	-	-	0.325–2.6
V	Setting accelerator	1.50 ± 0.03	2.75 ± 0.75	Beige	-	-	3–10
VI	Plasticizer/set retarding	1.14–1.2	7–9	Pink	<0.10	(−20)~(+80)	0.25–2
VII	Air-entraining	1.00–1.10	5–6	Brown liquid	<0.10	(−20)~(+80)	0.1–0.6

**Table 3:** Mix design for 3D printable mortars (kg/m^3^).

CODE	Cem.	GGBFS	Water	Microfiber	Clay	Type of Chemical Admixture *							Aggregate	
						I	II	III	IV	V	VI	VII	0–0.5	0–1
A0	680	136	285.6	1.82	2.45	0.88	8.16	4.08	4.08	8.16	4.08	0	333	675
A1	680	136	285.6	1.82	2.45	0.88	8.16	4.08	4.08	8.16	4.08	0.82	332	674
A1.5	680	136	285.6	1.82	2.45	0.88	8.16	4.08	4.08	8.16	4.08	1.22	332	673
A2	680	136	285.6	1.82	2.45	0.88	8.16	4.08	4.08	8.16	4.08	1.63	332	673

*: See Table 2 for the types of chemical additives corresponding to the numbers.

**Table 4:** Fresh and hardened 3D printable mortar test results.

CODE	Unit Weight (kg/m^3^)	Air Content (%)	Flow Diam. (cm)	Initial Setting Time (min)	Comp. Strength (MPa)
A0	2130	2.5	14.5	35	54.5
A1	1820	6.5	16.3	60	28.6
A1.5	1780	7.5	16.5	75	18.9
A2	1670	8.5	16.7	90	11.8

**Table 5:** Rheological properties of 3D printable mixtures.

*Code*	Yield Stress (Pa)		Bingham Viscosity (mPa.s)		Thixotropy (A_thix_)	Thixotropy
	Up	Down	Up	Down	Hysteresis Area (Pa/s)	Hysteresis Area (Pa/(s·cm^3^))
A0	262.2	131.8	37,232	47,697	A = 18,792	A_rel = 536.92
A1	155.4	68.3	26,457	26,959	A = 18,288	A_rel = 522.52
A1.5	99.0	50.9	26,343	24,606	A = 19,372	A_rel = 553.49
A2	116.0	59.4	31,110	26,523	A = 25,877	A_rel = 739.35

## Data Availability

The data presented in this study are available on request from the corresponding author.
